# Not by transmission alone: the role of invention in cultural evolution

**DOI:** 10.1098/rstb.2020.0049

**Published:** 2021-07-05

**Authors:** Susan Perry, Alecia Carter, Marco Smolla, Erol Akçay, Sabine Nöbel, Jacob G. Foster, Susan D. Healy

**Affiliations:** ^1^ Department of Anthropology, and Behavior, Evolution and Culture Program, 341 Haines Hall, UCLA, Los Angeles, CA 90095, USA; ^2^ ISEM, Université de Montpellier, CNRS, IRD, EPHE, Montpellier, France; ^3^ Department of Anthropology, University College London, London, UK; ^4^ Department of Biology, University of Pennsylvania, Philadelphia, PA, USA; ^5^ Université Toulouse 1 Capitole and Institute for Advanced Study in Toulouse (IAST), Toulouse, France; ^6^ Laboratoire Évolution et Diversité Biologique (EDB UMR 5174), Université de Toulouse, CNRS, IRD, Toulouse, France; ^7^ Department of Sociology, 264 Haines Hall, UCLA, Los Angeles, CA 90095, USA; ^8^ School of Biology, Harold Mitchell Building, University of St Andrews, St Andrews, UK

**Keywords:** creativity, cultural evolution, individual differences, innovation, invention

## Abstract

Innovation—the combination of invention and social learning—can empower species to invade new niches via cultural adaptation. Social learning has typically been regarded as the fundamental driver for the emergence of traditions and thus culture. Consequently, invention has been relatively understudied outside the human lineage—despite being the source of new traditions. This neglect leaves basic questions unanswered: what factors promote the creation of new ideas and practices? What affects their spread or loss? We critically review the existing literature, focusing on four levels of investigation: traits (what sorts of behaviours are easiest to invent?), individuals (what factors make some individuals more likely to be inventors?), ecological contexts (what aspects of the environment make invention or transmission more likely?), and populations (what features of relationships and societies promote the rise and spread of new inventions?). We aim to inspire new research by highlighting theoretical and empirical gaps in the study of innovation, focusing primarily on inventions in non-humans. Understanding the role of invention and innovation in the history of life requires a well-developed theoretical framework (which embraces cognitive processes) and a taxonomically broad, cross-species dataset that explicitly investigates inventions and their transmission. We outline such an agenda here.

This article is part of the theme issue ‘Foundations of cultural evolution’.

## 1. Introduction

During rapid environmental change, the success of hominid populations has depended on their ability to devise new fitness-enhancing behaviours that exploit novel aspects of the environment and are socially transmitted to conspecifics [1]. The extensive social transmission of novel behavioural variants is called innovation. Although humans are certainly an extreme case in their capacity to innovate, innovation is likely to be an adaptive strategy in many species. Our understanding of the evolutionary dynamics of such species will be limited, unless we attempt to document and model these innovation processes.

To explain innovation as a form of (Darwinian) cultural evolution requires (i) variation, i.e. the invention of novel behaviours or artefacts; (ii) heredity, i.e. the transmission of novel traits between individuals; and (iii) selection and other processes that can establish some traits as stable characteristics of a (sub)group [2]. Most research on the dynamics of cultural evolution has focused on transmission and selection; above all, on social learning and its mechanisms. In this paper, however, we emphasize the importance of investigating invention as a critical source of variation and thus a driver of cultural evolution.

As scholars of cultural evolution, we want to understand how natural and cultural selection act (at the level of the behavioural trait, the individual, the dyad, the group and the species) to produce varying types of cultural dynamics that lead to innovation (or not). The comparative method is a useful tool for elucidating such general principles—for understanding the evolution of innovative capacities generally, and for understanding the human case specifically. Species differ in their characteristics (demographic, ecological, psychological and morphological) and in the ease with which they can be studied for particular purposes.

In §2, we survey existing comparative and theoretical scholarship, which studies invention and innovation at multiple levels of analysis. Prior research on invention and innovation has been unequally distributed across psychological mechanisms, taxa, levels of analysis and methodologies. It has also largely neglected the cognitive-computational processes involved in producing inventions. As we show, the available data are too sparse and extant methodologies too poorly matched to the research questions to afford a cogent cross-species analysis of the reasons why species vary in their cultural capacities (particularly for invention).

After reviewing what is currently known about invention, and identifying major knowledge gaps and methodological flaws, we propose in §3 a set of promising research directions (observational, experimental and theoretical) that could transform our understanding of invention and innovation in the natural world.^1^ To make progress, the field needs a systematic effort by empirical researchers to collect data from a wide range of species, in dialogue with theoretical scholarship that connects computational-cognitive and evolutionary processes. Such data and theory are essential to unearth the general principles behind innovation as an adaptive strategy.

### (a) Definitions

Here, we define the terms we use in discussing the three distinct processes central to the study of innovation: the creation, transmission, and establishment of novel behaviour.

We define ‘invention’ as the creation of novel behaviour. Researchers of non-human animals often call these inventions ‘innovations’. We use the term ‘innovation’ to refer to inventions that succeed in diffusing widely through a (sub)group to become stable characteristics of that (sub)group.^2^ Another source of definitional confusion is that the word ‘innovation’ is used to describe both process (i.e. the transmission and establishment of an invention) and product (i.e. a behaviour that has been acquired by multiple members of a population through initial, individual invention and subsequent spread via social learning). We use ‘innovation’ to refer to process and ‘an innovation’ to refer to product.

Invention has been defined differently by different camps of researchers, according to the research question they are addressing. Some researchers cast their nets broadly, including any novel behaviour as an invention. Others impose additional restrictions, e.g. that the behaviour serve an obvious useful purpose, or impact the performer’s fitness. Sometimes it is stipulated that the behaviour must be something that a typical member of the species would not do under similar circumstances [6]; this parallels the stipulation in US patent law that a patentable invention could not be created by a ‘person having ordinary skill in the art’. Typically, a behaviour is not described as an invention if it is acquired via successful social learning.

To evaluate the selective pressures that influence processes of invention, it makes sense to use a broad definition. When an individual stochastically produces a behaviour it has never performed or seen performed before, we call that novel behaviour an invention. This definition excludes new behaviours acquired via social learning. It also excludes behaviours that occur naturally at certain points in development for all individuals, given particular environmental circumstances.^3^ It does include (i) novel behaviours produced by processes other than insight learning, (ii) behaviours that are creative but apparently useless or costly, and (iii) behaviours that are likely accidental the first time they are performed. Notably, we do not require inventions or innovations to be fitness-enhancing for individuals or groups.

We make no reference to cognitive processes in our definition because there are many ways something new might come about. Some inventions are produced by accident, when individuals fortuitously perform old actions in new circumstances, thereby producing new outcomes. Some inventions are generated by ineffectual social learning, when an animal fails to produce the observed behaviour and produces a new one instead. Other inventions are produced via deliberate problem solving, either trial-and-error learning or insight learning (i.e. solving a problem without trial-and-error, via mental rearrangement or restructuring of elements in a problem, perhaps based on past experience with some elements of that problem, resulting in a solution); this is important when old solutions fail in novel circumstances, such as the absence of some critical material. A specific evolutionary scenario that explains why some classes of individuals are more likely to invent than others is likely to apply to inventions generated by a subset of these cognitive processes. We therefore favour an inductive approach that starts with a broad definition of invention and attempts to explain patterns through some combination of cognitive process, life history, and ecological context.

## 2. What do we know about invention?

A comprehensive exploration of invention and innovation is multi-scale. It investigates how natural and cultural selection act on variation at the level of the behavioural trait, the individual, the dyad, the group and the species. It grounds these processes in ecological context. And it acknowledges that the link between selection and invention is mediated by the cognitive processes that generate inventions and lead to their diffusion and establishment.

We organize our discussion of the existing observational, experimental and theoretical literature around four levels of analysis: behavioural traits, individual characteristics, ecological circumstances and group characteristics. In doing so, we collapse some of the levels above (e.g. dyad and group), while weaving others (cognitive-computational processes) throughout.

### (a) What characteristics of behavioural traits make them more likely to be invented?

What can be invented by an organism? This question is critical but challenging to answer. An idea or behaviour could be ‘invented’ on multiple occasions at different times—or at the same time in different places [7]. The frequency with which a behaviour is re-invented might provide clues to how easy it is to invent.

The question of what can be invented has been addressed empirically and theoretically. Empirically, researchers have attempted to document the range of a species’ inventions—called the count method—and have categorized these into broad domains (e.g. capuchin monkeys [8]; orangutans [9]; chimpanzees [10]). A second empirical method gives humans and other animals problems to solve, usually a puzzle box with a reward inside, and asks how individuals invent solutions to the tasks (e.g. [11–13]). Both methods allow researchers to quantify and compare the behaviours that can be invented by an individual or a species, but the experimental approaches cannot be used to assess the range of inventions because possible behaviours are restricted by the task itself [14].

Researchers also face a methodological problem if they require that inventions have functional utility. When a novel behaviour is produced, its costs and benefits are likely unknown to the animal and the analyst. Some useless-looking behaviours are incorporated into individual and group repertoires. Take, for example, the human tradition of eating toxic cassava, which requires complex and non-intuitive processing to be edible, or the insertion of fingers in eye sockets as a bond-testing behaviour in capuchin monkeys [8]. The invention of these traditions probably involved a risky, apparently unpleasant novel behaviour. This would have looked like a mistake to a researcher coding the inventor’s behaviour in a short-term study. These examples challenge the notion that researchers can or should pre-judge the functional utility of behaviours.

It is more tractable to explore variation in the cognitive processes by which inventions are produced. Inventions can be produced by insightful problem solving, and many experiments are designed to explore that process. But inventions can also arise by serendipity, e.g. when individuals make mistakes in copying a target behaviour, or fortuitously perform a behaviour in a new context with desirable results [14]. Such inventions do not (necessarily) require insight learning, but they are no less new, are possibly useful, and may be transmitted to others.

Such discussions can be informed by existing theory on the computational and cognitive underpinnings of invention. Boden identifies three distinct creative processes [15]: the exploration of an existing framework; the combination of existing elements; and the ‘transformation’ of the space of possibilities (e.g. by adding new elements). Hofstadter and colleagues [16,17] offer more explicit formal models of the underlying computational substrate. In one model [17], concepts are represented in memory such that they afford exploratory variation about their central ‘theme’, with nearby variations being easier and more likely than distant ones. When the implications of such concepts intersect, they can be recombined into new concepts; combinations that are ‘close by’ are easier than those that are distant [18]. Although substantial translation is required to adapt these computational models to non-human animals, it is easy to see how they line up with existing notions like serendipitous discovery (mild variations on known behaviours or chance analogies between old and new behavioural contexts), trial-and-error learning (‘random’ variation or recombination) and insight learning (more selective variation or recombination).

Recombination is essential to human invention [16,19]. Rather than having to assemble the entire solution de novo, human inventors combine multiple existing solutions in novel ways [20]. The camera phone did not have to be built from scratch; inventors could put it together by modifying and combining well-understood technologies. Insofar as existing solutions are highly modular—with well-defined ways of linking them together—the inventor’s job becomes easier.

We know very little about the possibility of invention by recombination among non-human animals, where it might take the form of combining known behaviours in new ways or combining known behaviours with novel contexts or substrates. There is, however, some promising experimental work in some bird species that construct compound tools without trial-and-error learning, reinforcement or cueing; these findings suggest both recursive capacities (since tools are combined to produce new tools) and an ability to perceive when novel tools are required to accomplish a goal (e.g. [21]). Learning more about the abilities of wild animals to invent via recombination of existing behavioural elements requires documentation of individuals’ behavioural repertoires at a granularity rarely achieved in current studies. This is even more true for invention via transformation.

Cross-species differences in what characteristics of behavioural traits make them more likely to be invented are most likely driven by the underlying processes of invention. While it is probably the case, across species, that tinkering variation is more common than recombination, which is more common than transformation, species may vary substantially in their capacity to invent by these different processes, with some limited to variation and others capable of all three.

### (b) What characteristics of individuals make them more likely to invent?

The relationship between individual characteristics and propensity to invent has been addressed via theoretical models, literature reviews, experiments and observational studies (with captive and wild animals). We organize our survey of the existing literature around structural variables associated with differences in the propensity to invent: age, sex (the two sexes have different reproductive strategies and hence different time and energy budgets), dominance rank (a measure of resource monopolization ability), social network position, and personality. We note, however, that the mechanisms driving such associations likely depend on the underlying processes that drive invention, e.g. enhanced opportunity (via neophilia, network position or free time), persistence, or necessity (see §2c for more detail).

Cross-species comparisons face steep challenges. Although some meta-analyses have been performed (see §2c), interpretation is complicated by the methodological problems that we discuss below. When cross-species findings are inconsistent, this could be driven by methodological differences, or by differences in the way that structural variables link to underlying mechanisms across species. Firmer conclusions await the collection of more datasets designed specifically to answer these research questions using comparable methods.

*Age:* It is not clear how age affects invention. In a review of the primate literature, Reader & Laland [22] concluded that adult primates invent more often than do immatures, with the exception of chimpanzees (*Pan troglodytes*), which exhibited the opposite pattern. Kummer & Goodall [23] also claimed that invention in chimpanzees was more common in juveniles than in adults, though this claim was subsequently disputed [10]. In a meta-analysis covering mammals and birds, Amici *et al.* [24] also found that older individuals were more likely to invent, but there was no consistent methodology for defining inventions.

These comparative reviews share a deep difficulty: they are not based on reports from researchers explicitly measuring invention, and are susceptible to observer biases (such as for behaviours that look especially human-like or peculiar to the human eye). Larger literature reviews (e.g. [22,24]) do not always distinguish between invention as ‘success at solving a problem’ versus invention as ‘novelty.’ The former definition may be biased towards older (larger, stronger, more experienced) individuals and the latter towards younger ones.

Only one observational study in the wild has made a systematic attempt to record inventions during data collection, as they arise in a population, rather than using data collected for other purposes. Perry *et al*. [8] conducted a decade-long study of wild capuchin monkeys (*Cebus capucinus*) to measure rates of invention and innovation and determine the characteristics of especially inventive individuals. Older, more socially-central individuals were more likely to invent new forms of social interaction, while younger capuchins were more likely to invent new foraging-related behaviours and new ways of manipulating their environments, as well as their own bodies. Younger capuchins also exhibit a more diverse repertoire of actions when trying to open *Luehea* fruits [25], though it is not clear whether this is due to higher rates of invention in younger animals or experience-related pruning of inefficient techniques in older ones.

The findings from experimental approaches are, likewise, mixed. For example, juvenile red-fronted lemurs (*Eulemur rufifrons*) and chimpanzees (*Pan troglodytes*) were more likely to be the first to solve a two-action task [26], and to learn about new species of nuts to crack [27]. But older meerkats (*Suricata suricatta*) and captive callitrichids (seven species, from the genera *Leontopithecus, Callithrix* and *Saguinus*) were more likely to solve a novel task (extracting food from a puzzle box), perhaps because of their greater dexterity [11] and motor competence [28].

Depending on the species and type of task, either the enthusiasm of youth and/or the wisdom of age can lead to inventiveness. Future studies should make separate evaluations of age-related changes in the following contributors to inventiveness: (i) attraction to novel objects or situations (neophilia, tendency to explore), (ii) tenacity in problem solving, (iii) creativity in finding solutions (e.g. number of options tried), and (iv) physical strength and dexterity. This is consistent with our broader suggestion to focus on the cognitive and physical processes of invention.

It may also be the case that individuals of different ages actually learn differently. Gopnik *et al*. [29] argued that human children have more flexible and exploratory learning strategies than adults. As a result, children are better than adults at deducing unusual abstract causal principles from observations, whereas adults are less creative but more efficient in their learning strategies. Other researchers have claimed that young children are proficient social learners before they develop creative problem-solving skills, at least for tool use [30,31]. As Fogarty *et al*. [32] note, there are almost certainly age-related changes in particular types of learning skills that need to be taken into account, along with population structure, when developing models of cultural evolution. Agent-based models developed by Lehmann *et al*. [33] to better understand the circumstances that favour the accumulation of modifications over time (cumulative cultural evolution, CCE) examined the coevolution of life-history stages with the timing of use of social learning versus individual learning. They found that CCE is favoured when infants learn from non-parental adults (oblique social learning) while juveniles use a mixture of individual learning and learning from their peers (horizontal social learning).

*Sex:* Predictions regarding the impact of sex on propensity to invent stem from differences in body size (and hence competitive ability and free time) as well as differences in knowledge as a consequence of sex-biased dispersal. As with age, it is not clear whether females or males are more inventive, although in one meta-analysis of data on novel foraging tasks in 29 bird and mammal species, the larger-bodied sex was more likely to invent [24]—a finding that supports the ‘Free Time/Excess Energy’ hypothesis but not the ‘Bad Competitor’ hypothesis (see §2c). Considering the different life histories of males and females, one might predict that the dispersing sex would need to invent more than the sex that remains with kin in the birthplace, because the dispersing sex is more likely to encounter novel situations. When a disperser joins a new group, it may also need to learn behaviours from the new group members. It might also have to be inventive because it has less access to other individuals it might copy. The data are mixed regarding this hypothesis, however. For example, female red-fronted lemurs (the stay-at-home sex) and male meerkats (the leaving-home sex) were both more likely to solve an experimental task [11,26]. While the primate literature suggests that males may be more inventive than females [22], Perry *et al*. [8] found no differences between male and female capuchins across a decade of observations.

*Dominance rank:* The ‘necessity is the mother of invention’ hypothesis predicts that low-ranking individuals should be more inventive, while the Free Time/Excess Energy hypothesis predicts that high-ranking individuals, who have greater access to resources, will use their spare time to find new things to do [24]. To date, meta-analyses on invention in foraging tasks yield no evidence that dominance predicts inventiveness across species [24]. For example, low-ranking chimpanzees are sometimes more inventive than high-rankers [22], while the most dominant starlings (*Sturnus vulgaris*) participating in an experimental problem-solving task were the fastest to learn how to solve the task [34]. In wild capuchins (*Cebus capucinus*) and wild hyenas (*Crocuta crocuta*), invention seems unrelated to dominance rank [8,35].

*Social network position:* The position an individual occupies in its social network could foster opportunities for being inventive. There is a well-established relationship between network position (specifically, the spanning of ‘structural holes’) and invention in the literature on human invention [36]. In the animal literature, better-connected great tits (*Parus major*) and baboons (*Papio ursinus*) were more likely to find and use novel foraging patches, relative to individuals with more limited social connections [37,38]. Proximity to conspecifics might prompt certain kinds of object exploration, due to stimulus enhancement or social facilitation; however, a meta-analysis by Amici *et al*. [24] including 20 species of birds and mammals did not find a convincing relationship between proximity to others and propensity to invent, though the relationship was stronger in wild animals than in captive animals. The relationship between network position and inventiveness may be contingent on behavioural type: Perry *et al.* [8] found that more social wild capuchins were more prone to invent new social interactions, while less social individuals were slightly more likely to invent new foraging behaviours or ways of manipulating their own bodies.

*Personality:* Methods for studying animal personality are now well developed and there is considerable evidence of a role for personality in a range of animal decision-making [39]. The time is ripe to conduct more rigorous investigation of the impact of personality traits on propensity to invent and transmit innovations. Brosnan & Hopper [40] discuss five psychological factors that may limit innovative capacities, either in invention or transmission, by inducing individuals to stick with what they know rather than exploring novel options: neophobia (aversion to novel objects or situations), conservatism (not wanting to try new things), conformity (behaving like the majority), functional fixedness (being disinclined to use familiar objects in novel ways), and the endowment effect (a preference for objects/foods already in their possession rather than potentially more desirable objects not yet in their possession). Although the authors cite some evidence in favour of these ideas, the evidence is still scant and more comparative data are desirable. Stable inter-individual differences in proclivity to invent, as seen in foraging guppies, *Poecilia reticulata* [41], support a role for personality traits in inventiveness. Starlings (*Sturnus vulgaris*) that were quickest to feed in a novel experimental environment were also generally the ones who solved a task more quickly, which might suggest that boldness or exploratory proclivity promotes invention [34]. Male grackles (*Quiscalus lugubris*) that were less neophobic, more exploratory and more persistent were more likely to succeed in opening a box containing food [42], and the main predictor of success for wild hyenas in obtaining food from a puzzle box was diversity of techniques tried, with the primary inhibitor being neophobia [35]. Neophobia also seems to explain speed of problem-solving in raccoons (*Procyon lotor*): less neophobic and more persistent individuals were more likely to solve a puzzle-box task [43]. Horses (*Equus caballus*) that were more active, more tenacious, and better at inhibition control were better able to feed from a novel feeder [44]. Amici *et al*.'s [24] meta-analysis of 38 studies of foraging tasks in 20 species of birds and mammals showed that individuals that are more explorative, neophilic and (to a lesser, non-significant extent) persistent were more prone to invent; exploration more strongly predicted invention in captive rather than wild animals.

On this account, propensity to invent is (in part) an emergent consequence of personality traits like persistence and neophilia. Problem-solving experiments with several species of birds provide further support for this hypothesis [12]. Certain aspects of morphology also enable more diverse ways of manipulating the environment. Interactions between cognitive, morphological and personality/motivational traits are thus likely to result in both individual and species differences in rates of invention [45,46].

The literature on humans is broadly consistent with the provisional findings from the comparative literature, with plenty of speculation as to the types of personality traits likely to promote invention and innovation. Sternberg [47], summarizing his life work in this area, speculates that all of the following traits may play a role in promoting innovation: willingness to overcome obstacles (possibly akin to perseverance), willingness to take sensible risks, tolerance of ambiguity, and tendency to seek opposition (which might be seen as contrariness or as anti-conformity). Simonton [48] emphasizes some overlapping traits, including independence, anti-conformity, openness, ‘behavioural and cognitive flexibility and boldness’; because he views human creativity as a Darwinian process in which successful inventions arrive through variation and selective retention [49]—a point of view that goes back to William James [50]—Simonton emphasizes the role of these traits in ‘the production of ideas both numerous and diverse’.

Mood in humans can affect inventiveness. As a transient emotional state, mood is not the same as personality, but some personality types might be more prone to particular moods. People with positive moods were more creative than those with neutral moods [51] and, in general, individuals more sensitive to positive rather than negative outcomes were more creative. Although emotions are increasingly studied in non-human animals, their role in inventiveness has not been carefully examined.

These ideas resonate with computational theories of creativity and invention. Such theories emphasize novelty-seeking, whether through the recognition (and avoidance) of experiential ‘ruts’ [17] or through intrinsic reward from encountering new experiences that can be profitably ‘compressed’ through learning [52]. Many of the traits cited by Sternberg and Simonton (as well as the non-human literature) reflect the novelty-seeking dispositions described by formal theory; even the mood results can be understood through the interplay of intrinsic reward (from novelty-seeking behaviour) and extrinsic reward (food, threats, and other ‘rewards’ from the environment).

It is likely that age, learning strategy and personality traits interact to produce variations in inventiveness. Natural selection could have favoured both (i) within-species shifts in personality traits or attitudes relevant to learning strategies across different life-history stages, and (ii) different timing of these shifts across species that vary in their life-history strategies [25]. For example, younger capuchin monkeys (*Cebus capucinus*) are less neophobic and more playful, creative, curious, opportunistic and active than older monkeys; they are also more prone to attend to foraging conspecifics [25]. These traits have obvious implications for propensity to invent and copy novel behaviours. Formal theory, combining models of biological and cultural evolution with computational models of the inventive process, could sharpen these hypotheses and guide subsequent empirical research. Indeed, theory is essential to select the most fruitful possibilities, given the combinatorial explosion of species and factors involved.

### (c) What circumstances make inventions more likely?

Several contextual factors have been suggested as drivers of invention: (i) necessity (e.g. the most inventive individuals have little access to resources because they are subordinate, in poor body condition, and/or too young to compete effectively); (ii) access to opportunities (e.g. higher encounter rates with particular resources promote attempts to exploit these resources [53], as with new forms of tool use); and/or (iii) free time/energy (e.g. the most inventive individuals are higher ranking, in better body condition, and of the larger sex, because they need to spend less time foraging and can assume higher risk foraging strategies [23,24]). Of course, these are not mutually exclusive possibilities, and all have received mixed support. Amici *et al*. [24] attempt to distinguish the ‘Bad Competitor’ hypothesis (which they equate with ‘necessity’) and the ‘Excess Energy’ hypothesis. Their analysis did not convincingly support either hypothesis, as there were no clear differences related to rank or body condition. Furthermore, older animals were more prone to invent in their meta-analysis, but the authors predicted that younger individuals should be more inventive under both hypotheses. These authors argue that their finding that the larger-bodied sex was more inventive supports the ‘Excess Energy’ hypothesis; however, the assumption that the larger sex needs to spend less time foraging seems debatable, as the larger sex may (i) have a higher metabolic rate or (ii) have a reproductive strategy requiring more time spent in social competition relative to the smaller sex.

These methodological difficulties could be resolved by measuring invention, body weight, competitive ability, food intake, and activity budgets more explicitly, in a way that permits greater cross-study consistency; the variables used in this meta-analysis [24] were, of course, measured for different research agendas. It would be particularly desirable to measure ‘free time’ independently from ‘excess energy’ and competitive ability; these variables are not necessarily correlated. Dominants, by definition, have higher competitive ability, but they may have less free time because they devote more time to servicing social relationships, compared to subordinates. And alpha males may require extreme amounts of energy to maintain their bodies in good fighting condition to defend their positions. We also note that these hypotheses could be extended from comparisons within species to comparisons between species or between ecological contexts; some accounts of hominid inventiveness, for example, appeal to both our low competitive ability and substantial variability in the ancestral environment.

### (d) What characteristics of groups make inventions more likely to spread?

Innovation requires more than invention; novel behaviours must then spread and stabilize in a (sub)group. How group characteristics (and the structure of social networks) affect the spread of inventions depends on the type of behaviour, which in turn influences the mode of transmission. Some inventions, like internet memes or simple behaviours, are easily transmitted, and spread like an infectious disease (simple contagion). Other inventions (e.g. an elaborate food extraction technique) are less easily transmitted; they follow a ‘complex’ contagion dynamic, where a single exposure is not sufficient for acquisition [5,54].

*Social networks:* In simple contagion, information may spread more rapidly through dense rather than sparse networks. In dense networks, individuals interact more with each other, which increases opportunities to observe others [33]. For example, information was shared more rapidly on the social network site Digg, compared to the less dense Twitter [55]. Highly clustered networks are predicted to impede information flow, as information gets ‘trapped’ in local clusters [56].

If transmission follows complex contagion dynamics, however, an individual’s probability of adopting novel traits is higher if she receives social reinforcement from multiple neighbours. Here, clustering is beneficial. In a study on the adoption of health behaviour, new behaviours were more readily and widely adopted in clustered networks than in random networks [57]. Conversely, Smolla & Akçay [58] show that with complex contagion dynamics, dense networks coordinate on a few common traits, which impedes the spread of novel traits generated by individual learning.

Individuals may preferentially associate with those sharing similar phenotypes, resulting in positive assortment or ‘homophily’ at the network level. This can affect transmission. Homophily may preclude some individuals from obtaining social information because the individuals who are more likely to generate information [59] may not associate with naive ones [56]. Conversely, negative assortment (heterophily) may facilitate the transfer of information between information generators and non-generators. Thus, the propagation of information through a social network could be limited by positive assortment of information-generating phenotypes, and enhanced by negative assortment. A similar mechanism plays out in human social networks; because advantaged individuals are more likely to adopt a new behaviour, and because they tend to associate with each other, beneficial behaviours tend to spread to already advantaged individuals [60]. This mechanism can be subtle, however; in an experimental study, homophily promoted the adoption of a novel health behaviour in an online social network [61]. This might be because humans are more likely to be influenced by others sharing similar traits—a version of directed social learning—and limited homophily across a mixture of characteristics [61] facilitates directed social learning.

These results suggest that variation in natural (and experimental) networks can inform which network structures promote or retard information flow, holding individual characteristics constant. Such research is most productive in dialogue with formal models that predict transmission in real (and experimental) populations of humans and non-human animals [54].

*Age composition of groups and populations:* Age is a critical variable, affecting how much individuals learn socially (versus asocially) and whom they learn from. Despite this, there has been little empirical or theoretical work on how age variation in a population affects the spread and maintenance of innovations.

One class of models [33,62] considered the evolution of when and how long individuals learn socially versus individually, and the consequences of these strategies for the accumulation of culture across generations. They show that two factors affect the evolution of a learning schedule that can sustain innovations across generations: the efficiency of different kinds of learning, and the trade-off between learning or inventing new behaviours versus exploiting them.

Another class of models investigates the spread or decline of socially learned behaviours in age-structured populations with age-dependent learning rates. Fogarty *et al*. [63] use this approach to understand the impact of social learning on fitness-changing behaviours (e.g. obtaining more education and having fewer children). They found that transmission of such behaviours between unrelated individuals can cause demographic transitions in which the age-structure rapidly changes. These models take into account the reciprocal feedback between the age-structure of a population and the traits that change it; such feedback can have profound effects on the spread and maintenance of cumulative culture. Another agent-based model of cumulative (but demographically neutral) culture [64] endows individuals with different learning rates as well as bias in whom they learn from. In that model, populations accumulate more cultural traits if individuals live longer (because they have more learning opportunities), but the populations have lower rates of cultural change. Interestingly, biasing social learning towards older individuals, even if they are more conservative, results in a rate of cultural change similar to or even higher than when individuals meet at random. This is because older, more experienced individuals are better cultural models.

These disparate modelling approaches all show that the age-structure of the group—and how individuals of different ages learn from others—can profoundly affect the spread and maintenance of cultural traits. At the same time, this topic remains underexplored; for instance, we do not yet know how age assortment in social networks affects the spread of innovations, or how age-dependent invention propensities interact with demographic structure to determine long-term dynamics of cumulative culture.

## 3. What do we not know about invention and innovation?

Our survey of the existing literature on invention and innovation revealed substantial methodological and theoretical gaps. This is unsurprising, given the longstanding focus on social learning in the study of cultural evolution. In this section, we discuss some of those gaps and suggest ways to fill them.

### (a) Gaps in the experimental literature

To understand the role of invention and innovation in biological and cultural evolution, we need to answer basic questions across a range of taxa: what gets invented? By whom? In what circumstances? And does it ‘stick’? To answer these questions, it is essential to ground research on animal invention (and innovation) in solid natural history. Until now, most empirical research on inventions has suffered from one or more of three flaws:

— Observational work on behavioural novelty has been based on retroactive interpretation of data collected for other purposes. It is subject to human memory and research biases about which behaviours are recorded and interpreted as novel.
— In experimental studies, a novel task is presented to the animals by a researcher who has explicitly designed the task to be abnormal enough (for that species) that the task is definitely novel; participants often need to be trained to engage in the task. Invention in these studies is often defined as being good at solving the task in the way the human researcher intended. Such approaches do not permit the research subjects to express their full range of creativity.
— Both observation and theoretical studies are relatively divorced from formal models of the inventive process, which could inform study design and reconcile competing hypotheses.

How could the field move forward? Determining rates of invention is perhaps the greatest empirical obstacle to research on inventions, closely followed by characterizing the (changing) space of possible inventions (which is necessary to assess what characteristics make inventions more or less likely). Systematically cataloguing the building blocks of species’ inventions (i.e. the behaviours that could be combined in order to create new ones) would help address the issue of what is ‘invent-able’. Lack of systematically collected data on inventions also hampers our understanding of how species differ in their inventive abilities, and how often particular creative products are independently invented.^4^

Progress requires the collection of longitudinal datasets that systematically record the fine details of species-typical behaviour, along with any novel behaviours observed. Such data have transformed the study of human invention in science and art [67,68]. It is essential that we collect similar data for large numbers of individuals, social groups and species.

The only naturalistic study of invention that has attempted systematic documentation of entire repertoires focuses on ten white-faced capuchin groups [8]. In this 10 year study, a large staff of observers (previously trained to identify all elements of the species-typical repertoire) was trained to report any novel behaviour in detail, across behavioural domains. A observer with 26 years of experience on the study population evaluated each observation, terming it an invention only if (i) it had not been seen previously in that individual or group during the lifetime of the putative inventor in the 10-year period, and (ii) the behaviour was absent in the repertoires of at least some groups.

By collecting such data, along with association patterns and gaze directions, researchers can infer which behaviours are new and which are socially learned or readily invented independently. In addition to characterizing the inventive space, long-term studies collect detailed data about the kinship, age, association patterns, personalities and relationship histories of the individuals. This enables researchers to answer questions about the qualities of individuals that promote inventiveness. These studies can also gather consistent data about competitive ability and activity budgets, allowing them to adjudicate between hypotheses about inventive context (e.g. necessity versus excess energy).

One challenge in defining new behaviours is deciding how finely to parse behavioural sequences. All behaviours (novel and not) are constructed from basic ‘building blocks’: motor patterns that are part of a species-typical repertoire. Invention occurs in the application of these building blocks to new contexts, or their combination into new sequences. If coding is sufficiently fine-grained (e.g. all motor actions and the objects and contexts they are applied to), it should be possible to make more objective decisions about what is novel. Advances in machine learning may accelerate such coding, ultimately mitigating observer bias [20,69].

Another challenge faces studies of the ‘invent-ability’ of behaviours. We cannot fully imagine or predict the universe of ‘behaviour space’ for behaviours that have not yet been invented but could be (in theory). We can, however, ask what kinds of behaviours are likely to be invented (and what properties of individuals make them more likely to invent certain kinds of behaviours), by documenting all the behaviours that have been witnessed. We can then assess their relative invent-ability by noting how often they appear in individual and group repertoires, taking into account exposure to appropriate contexts for displaying these behaviours. This approach could also address more specific questions, like: (i) given that a specific motor pattern has been performed in one context, what is the likelihood that it will be performed in another context? and (ii) given that an individual has performed step one of a (plausible) behavioural sequence, what is the probability that it will perform step two and step three? These data can be used to inform modelling approaches, including detailed models of the computational processes of invention.

Standard techniques for representing the dynamic structure of invention space in human science or technology (reviewed in [18]) could be used to represent behavioural repertoires and quantify the novelty of particular behaviours. Behaviours could be characterized by the presence or absence of different building-blocks, and the novelty of behaviours quantified by their distance from typical ones, in an appropriate metric [18,70]. This approach could distinguish incremental variations on existing behaviours from more substantial recombination—or the more radical transformation of invention space [15,17,20]. Invention space could alternately be represented by a network, in which building blocks are linked when they are observed in combination; the novelty of a particular behaviour is characterized by its structural position, e.g. whether it combines building blocks that have not been combined before, whether it introduces a new building block, or whether it combines building blocks from distinct behavioural clusters [65]. Similar techniques could be used to represent the way that distinct individuals combine specific behaviours in different contexts (paralleling the representation of scientists combining particular chemicals and methods to study certain diseases [66]). These rich representations can serve as input to models predicting the probability of behaviours being combined, or transferred across contexts, by particular individuals; they would be valuable input for data-driven models of underlying cognitive processes. They also connect to perhaps the biggest gap in our knowledge of social transmission amongst non-humans: whether individuals ‘prefer’ to learn some inventions over others—i.e. whether and how the particular characteristics of inventions affect their subsequent transmission, as in Rogers [5]). This is an essential step to a broader understanding of innovation in the animal world.

### (b) Gaps in the theoretical literature

New inventions do not appear randomly in the space of all possible inventions; there is structure in how that space is (stochastically) explored. As formal models of creativity [17] suggest, inventions that are close to existing behaviours are easier to create [18]. Some behaviours may also be easier to combine than others (and individuals may vary in the ‘combinability’ of their representations). Models of cultural evolution have paid very little attention to these issues, treating the space of possible inventions as unstructured. Among the few exceptions is a paper by Lewis & Laland [71], in which the authors distinguish between inventing completely new traits or tools, combining existing ones, and modifying them. This model abstracts away from individual, dyad, and network-level dynamics, and only considers the long-term dynamics of cumulative culture in a population. A more recent paper by Smolla & Akçay [58] imposes a simplified structure on the space of cultural traits, in which each trait builds on a single precursor. This framework can be extended to allow individuals to combine existing traits, or for a single trait to give rise to multiple ‘descendants’. By varying the probabilities of such events [71] and the structure of the population [58], we can ask how cumulative culture evolves in different inventive spaces.

Another interesting direction is to develop models of individual and social learning in more realistic dynamic invention spaces. Foster *et al.* [18] provide a possible foundation for this approach. They systematize several measures of novelty for patents, based on different representations of the space of existing knowledge and different models of the inventive process. They show that novelty measurement depends on appropriate, domain-specific models of invention, building a connection to more abstract models [16,17,52]. Such techniques for representing invention spaces and modelling inventive processes can be built into individual and social learning models to determine the probabilities of inventing new behaviours and the ease with which they are transmitted socially.

Substantial research on the diffusion of innovations in human groups has examined the characteristics of individuals who transmit and maintain inventions—either from the point of view of the learner, the model, or their relationship [5]. There is strong empirical evidence for inter-individual differences in motivation, capacity and opportunity for cultural learning (for a review, see [32,72]). Nevertheless, theoretical work on individual and social learning has largely neglected such differences. Some studies have varied the propensity for individual versus social learning, or let individuals with different social learning strategies compete (e.g. [73,74]). But there is more to individual variation than learning propensity. Future models should explicitly incorporate age structure, sex, dominance, kinship and personality as possible factors influencing attention and cultural learning. This will make models more complex, but this complexity is needed to better understand how individual differences and preferences interact to shape the diffusion of innovations. Turning to the role of social structure, some research has been conducted on the factors affecting transmission within a dyad. That said, empirical studies that look at how new behaviours or information spread along a network as a function of the distribution of individual traits remain rare, beyond humans. Likewise, most theoretical models assume that new behaviours appear in random individuals (akin to mutations), although we know that different classes of individuals might invent at different rates, as discussed in §2b. The location of such individuals in a network, their connectedness, as well as the correlation between individual traits over a network, will affect whether, and how, newly invented behaviours spread in a population. These questions remain largely unstudied, limiting our understanding of how network structure, diversity of individual traits, and their distribution over the social network affect the dynamics of innovation and cultural evolution.

## 4. Conclusion and future directions

Understanding the prevalence and drivers of innovation across the history of life requires an ambitious research agenda, with a renewed focus on invention. We cannot currently state how common innovation is as an adaptive strategy, because we cannot accurately quantify invention, the social learning of novelties, and their establishment as parts of group culture. Some species (e.g. cephalopods) may be extremely inventive as individuals, but have little capacity (or use) for social learning. Others may be less inventive individually, yet able to transmit and stabilize the rare inventions that do occur. Humans are quite good at both. Are other species?

To discover the evolutionary principles that shape invention and innovation, it is necessary to collect data from multiple, strategically chosen species. Comparative data are scant, particularly naturalistic data in which the animals themselves determine the problems to be solved. They are collected with such diverse methodologies that it is difficult to combine them into a credible comparative analysis. Available studies, which primarily target individual characteristics promoting invention, yield different answers within and between species. Clearer results will be obtained when the scientific community has obtained more data, using more stringent definitions and methodologies like those described here, and considered variation at the level of behaviour, individual, context and group, preferably integrating these levels of analysis. The roles of age, personality and social network structure in invention are particularly understudied (both empirically and theoretically). The few models of cultural evolution that take age or life history into account seem to indicate that learning strategies shift over time; it is thus critical to incorporate age structure into these models. Richer models are needed to guide empirical work through the combinatorial thicket of possibilities, including a robust engagement with computational models of the invention process.

Although field (and captive) experiments will always play an important role in understanding certain aspects of cultural evolution (especially mechanisms of invention and learning), they cannot substitute for careful collection of natural history data on behavioural variation of wild animals making choices in their natural environments. Longitudinal field studies—particularly those employing consistent data recording methods across decades—have a particularly important role to play in documenting how behavioural repertoires for individuals and groups change over time, in accordance with natural ageing processes, changes in group composition, and ecological changes. Such studies will provide opportunities to ground-truth models of cultural evolution that make predictions about the rates at which inventions will rise and spread under different assumptions about the network structure and demographic characteristics of groups.

Although there has been recent progress in building theoretical models of the invention and transmission of behaviours [58,71], most models still treat invention as a blackboxed, random process like genetic mutation. In parallel to the enriched treatment of biological variation in the extended evolutionary synthesis [75], models should incorporate variation at the level of the trait, individual, dyad, group structure and environment. More fundamentally, these simple models typically treat invention as incremental adjustments to existing behaviours. They should allow more complex recombination of behaviours and/or contexts, and draw on formal models of computational creativity [16,17,52] as well as data-driven work on human invention [18,68].

In this review, we have focused on models of invention, but transmission is critical to establishing inventions in behavioural repertoires. We know that individuals differ in their rate of invention and the inventions they create. We also know that the network position of individuals affects the probability that inventions will spread, becoming innovations. It is essential to develop models of invention and social learning that take structure into account—the dynamic structure of inventive space, and the dynamic structure of social networks.

Empirical data should guide the formulation of such models. Three methodological challenges slow empirical work on invention and its role in cultural evolution: (i) documenting behavioural repertoires (individual and group) and their change over time; (ii) documenting invention rates, and distinguishing between independent inventions and socially learned adoptions of traits; and (iii) documenting what proportion of ‘invention space’—i.e. combinations of behavioural elements and objects in routinely encountered ecological settings—is occupied by a particular individual or species and how that changes over time. Solutions to these challenges will greatly speed progress at the intersection of theoretical and empirical research on cultural evolution.

It is not by transmission alone that cultural evolution occurs. Invention provides the behavioural novelty on which cultural evolution operates. Until we invent the methods and models for studying this essential process, our understanding of cultural evolution will be partial at best.

## Footnotes

1 Space restrictions prevent us from thoroughly reviewing the state of the literature regarding diffusion and establishment of inventions in detail, but we describe some findings and also some gaps in the social learning and cultural evolution modelling literature relevant to determining (i) which behaviours spread and become established in group repertoires, and (ii) how population structure affects information transmission and accumulation.

2 In doing so, we essentially follow the tradition of Schumpeter, who distinguishes mere novelties from those that change the ‘production function’ of society [3,4], rather than Rogers, who uses innovation to refer to ‘an idea, practice, or object that is perceived as new’ [5].

3 It does so through our stipulation that the production of the behaviour is stochastic, i.e. involves some contingency.

4 Of course, it is not possible to anticipate specific inventions that introduce new components, though longitudinal data can be used to estimate the rate of these ‘jumps’ [65]. Despite the combinatorics, human invention is surprisingly predictable on short time scales [66], and the same is likely true for non-humans.

## Data accessibility

This article has no additional data.
